# Extranasopharyngeal angiofibroma of the sinonasal tract: A systematic review

**DOI:** 10.1007/s00405-025-09985-7

**Published:** 2026-02-16

**Authors:** Fabio Portella Gazmenga, Jochen P. Windfuhr, Fabio Lau, Mariana D.C. Toro, Eulalia Sakano

**Affiliations:** 1 Department of Otolaryngology, Head and Neck Surgery, University of Campinas, Campinas, Brazil; 2 Department of Otorhinolaryngology, Plastic Head & Neck Surgery, Kliniken Maria Hilf Mönchengladbach, Mönchengladbach, Germany

**Keywords:** Extranasopharyngeal angiofibroma, Sinonasal tract, Vascular tumors, Juvenile nasopharyngeal angiofibroma, Sinonasal tumor

## Abstract

**Purpose:**

Extranasopharyngeal angiofibromas of the sinonasal tract (ENA-SNT) are exceptionally rare tumors that remain poorly characterized in the literature. We aim to systematically review and synthesize the clinical characteristics, management, and outcomes of ENA-SNT.

**Methods:**

This review adhered to PRISMA guidelines. A literature search was conducted across major databases, including PubMed, EMBASE, Web of Science, and Google Scholar. Articles that reported ENA-SNT cases in humans were eligible for inclusion. Data extraction and quality assessment were independently performed by two reviewers.

**Results:**

A total of 145 studies were included, comprising 163 patients. The mean age was 28.8 years (range, 0–78), with a male-to-female ratio of 2.1:1. The nasal septum (33.3%), inferior turbinate (16.6%), and maxillary sinus (16.0%) were the most frequent tumor sites. Common symptoms included nasal obstruction (73.0%) and epistaxis (68.1%), often in combination (55.2%). Angiography demonstrated no hypervascularity in 23.1% of cases; when present, the internal maxillary artery supplied the tumor in 96.1%. Preoperative biopsy caused brisk bleeding in 44.5%. Surgery was the primary treatment in 94.4% of cases, with a recurrence rate of 5.3% and an average time to recurrence of 3.8 months. Intracranial and orbital involvement were rare: 1.84% and 2.45%, respectively.

**Conclusion:**

This review supports ENA-SNT as a clinical entity distinct from juvenile nasopharyngeal angiofibroma. Symptoms develop more rapidly, but the tumor is less aggressive, less vascularized, and has a better prognosis. ENA-SNT can occur across all age groups and in female individuals. Surgical resection is the treatment of choice, with low recurrence rates.

## Introduction

Angiofibromas are rare, highly vascular, benign tumors that account for less than 0.5% of all head and neck tumors [1]. Juvenile nasopharyngeal angiofibroma (JNA) is the most common clinical form, typically originating near the sphenopalatine foramen and primarily affecting male adolescents. Even more rarely, angiofibromas can originate outside the nasopharynx. These are known as extranasopharyngeal angiofibromas (ENAs) and are considered a separate entity due to their distinct clinical and epidemiological features [2]. Such characteristics can vary depending on the tumor’s specific location within the head and neck. Currently, there is limited information on the characteristics of ENAs that originate from the sinonasal tract (ENA-SNT), and no published systematic review has focused specifically on these cases. Given the limited data on this atypical presentation, it is crucial to compile and critically evaluate the available evidence to gain a deeper understanding of the disease.

This systematic review aimed to identify all reported cases of ENA-SNT and describe their clinical and epidemiological features, management approaches, and patient outcomes.

## Methods

This systematic review followed the Preferred Reporting Items for Systematic Reviews and Meta-Analyses (PRISMA) guidelines [3]. 

### Search strategy

A literature search was conducted across major databases, including PubMed, EMBASE, Web of Science, and Google Scholar. The search encompassed articles published from database inception to April 25, 2025. Furthermore, the reference lists of key prior reviews on ENAs were searched to identify additional eligible studies.

Database searches were conducted using the following strategy: (extranasopharyngeal angiofibroma) OR (nasal angiofibroma) OR (septal angiofibroma) OR (septum angiofibroma) OR (sinus angiofibroma) OR (maxillary sinus angiofibroma) OR (maxilla angiofibroma) OR (ethmoid angiofibroma) OR (sphenoid angiofibroma) OR (frontal angiofibroma) OR (turbinate angiofibroma) OR (nose angiofibroma) OR (atypical angiofibroma).

### Inclusion criteria

Articles that reported histologically confirmed ENA-SNT cases in humans, despite of the study design, were eligible for inclusion in this review. Given the extreme rarity of this disease, no language restrictions were imposed. Articles published in languages in which the authors are not fluent were professionally translated for review.

### Exclusion criteria

Reports that only provided an abstract (i.e., full text not available), cases of JNA, reports where ENA-SNT could not be confirmed, individual cases previously published in another report or series, and low-quality reports were excluded.

### Study selection and data extraction

Two authors independently screened all titles and abstracts for eligibility, with disagreements resolved by a third independent moderator. The full text of all eligible studies was reviewed, and the extracted data included participants’ characteristics, disease clinical features, management and intervention details, and patient outcomes.

### Quality of studies and bias assessment

The methodological quality of the included studies was assessed using a tool adapted from the Joanna Briggs Institute (JBI) assessment tool for critical appraisal of case series and case reports [4, 5]. Studies were rated as having low, moderate, or high risk of bias.

### Data analysis

Given the descriptive nature of this review, we used descriptive statistics to report clinical features. Continuous variables are presented with their means, medians, and standard deviations (SD), while dichotomous variables are expressed as frequencies and percentages. An illustrative map was generated online at mapchart.net and later adapted to our specific needs. Graphical presentations were plotted in GraphPad Prism Version 8.0.0 for Mac (GraphPad Software, Boston, MA; www.graphpad.com).

## Results

### Study characteristics and quality assessment

**Fig. 1:**
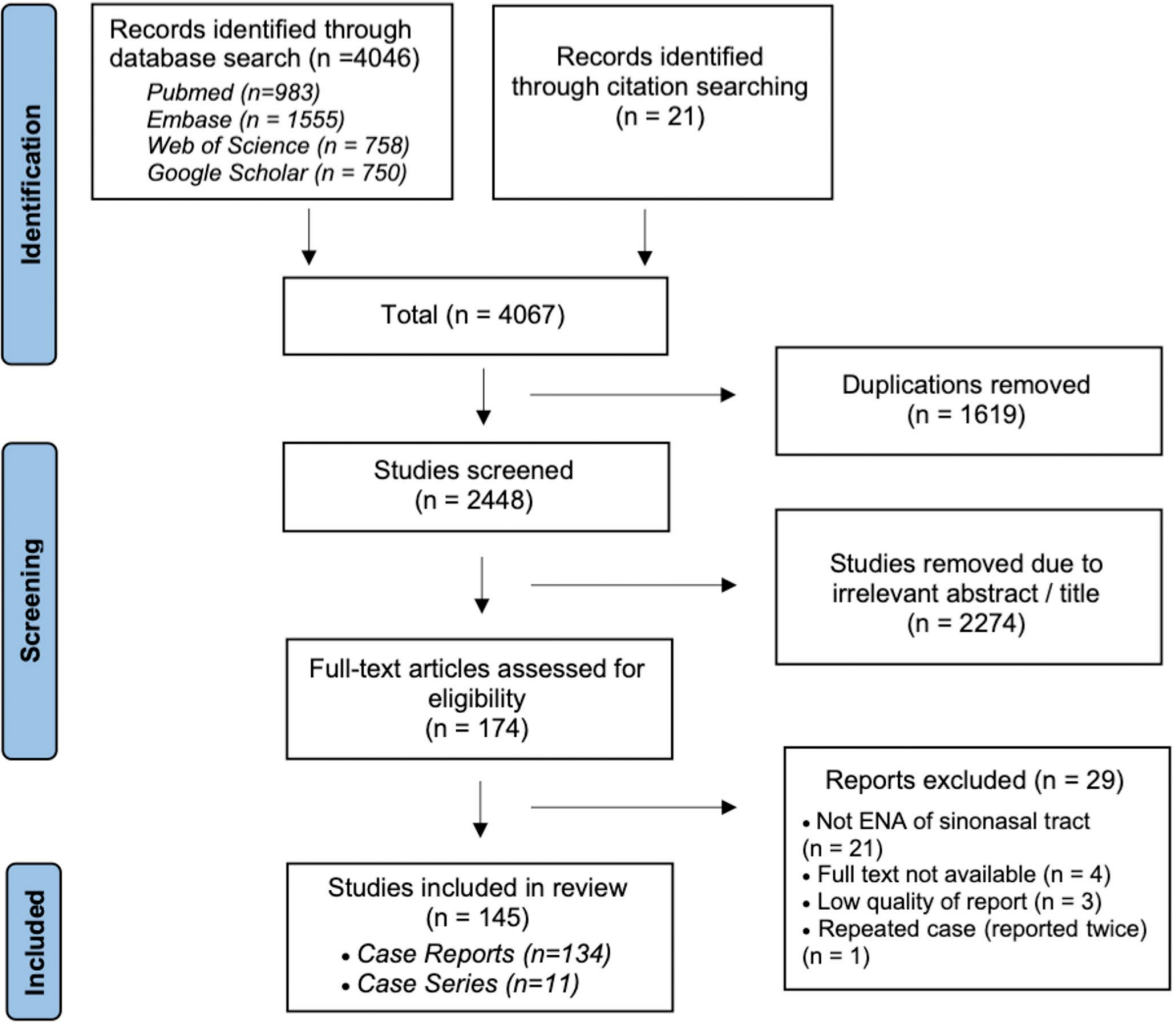
Preferred Reporting Items for Systematic Reviews and Meta-analyses (PRISMA) flow diagram

A PRISMA flow diagram is presented in Fig. 1. The initial search identified 4067 records, 2448 of which remained after removing duplicates. Following title and abstract screening, 2274 articles were excluded due to irrelevance. The full texts of the remaining 174 articles were reviewed, and 29 were excluded: 21 did not report on ENA-SNT, 4 lacked full-text availability, 3 were identified as low-quality reports, and 1 described a previously published case. Therefore, 145 studies were included in this review, comprising 134 case reports and 11 case series, which collectively encompassed a total of 163 cases of ENA-SNT.

All 163 cases met the satisfactory quality criteria based on the adapted JBI assessment tool for case series and case reports [4, 5]. Of these 145 studies, 89 (61.38%) were rated as having a low risk of bias, 50 (34.48%) as having a moderate risk, and 6 (4.14%) as having a high risk. A detailed description of the quality assessment for each study is provided as Supplementary Material 1.

**Fig. 2:**
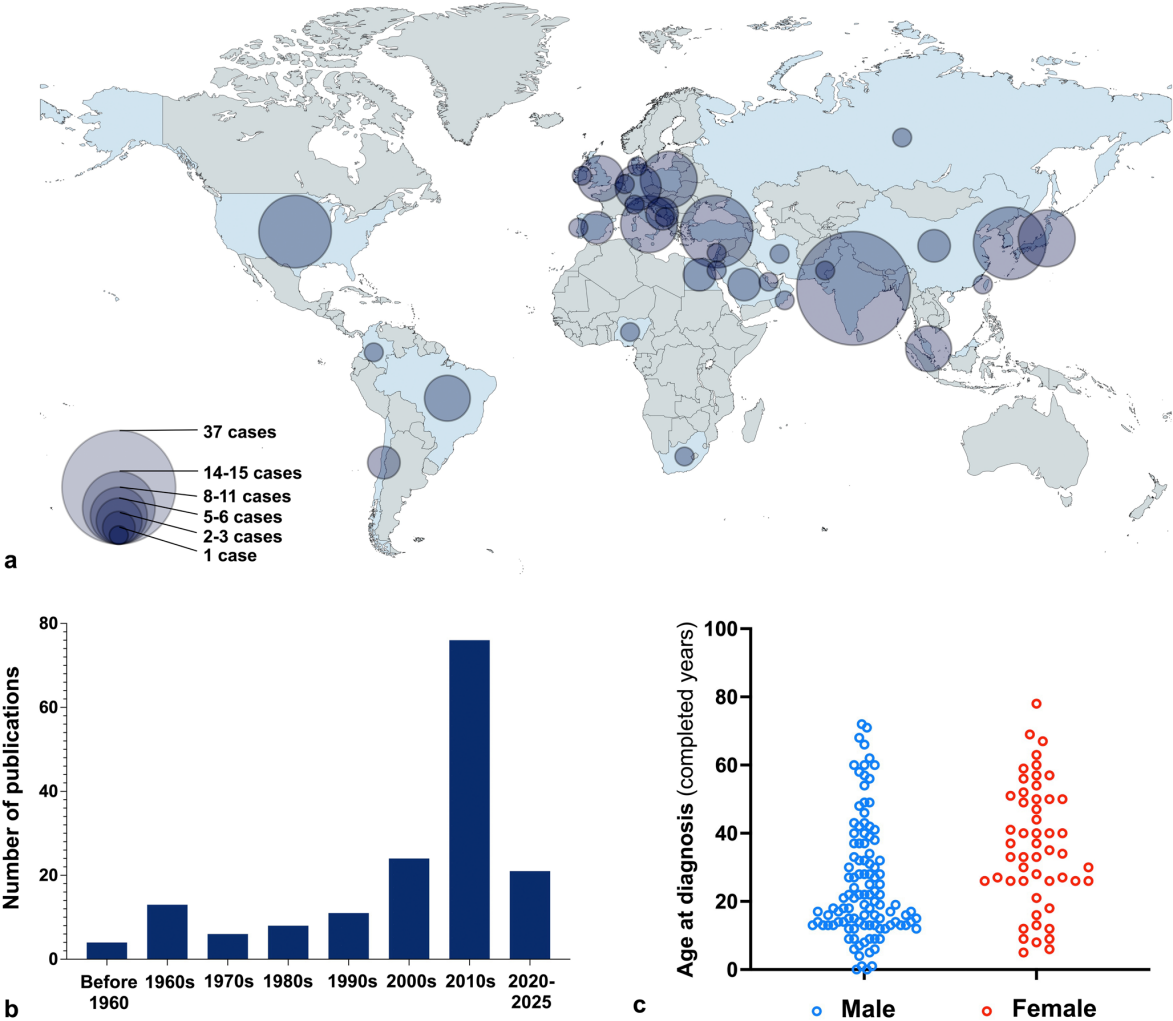
**A**, geographical distribution of reported cases. **B**, publication timeline of the included articles, by decade. **C**, distribution of individual cases according to age and gender. Each circle represents one case

Analyzing the country of origin of the included studies, 56.44% were from Asia, 27.00% from Europe, 13.50% from the Americas, and 3.06% from Africa. Specifically, India contributed the largest number of reports (*n* = 37; 22.70%), followed by Turkey (*n* = 15; 9.20%), and South Korea and the United States (both with *n* = 14; 8.58%). Figure 2a illustrates the geographical distribution of reported cases of ENA-SNT.

Figure 2b provides the publication timeline for the included articles.

### Patient characteristics

Figure 2c presents the distribution of the cases individually according to age and gender. Patient age ranged from 0 to 78 years, with a mean (SD) age of 28.84 (18.1) years and a median age of 26 years. There were 110 men and 52 women, with a male-to-female ratio of 2.1:1. The gender was not specified in 1 case.

Regarding tumor laterality, 84 cases involved the right side, 75 involved the left side, and 3 were bilateral. The side was not specified in 1 case.

### Tumor site

The nasal septum was the most common site (*n* = 56; 33.35%), followed by the inferior turbinate (*n* = 27; 16.56%), maxillary sinus (*n* = 26; 15.95%), ethmoid (*n* = 18; 11.04%), nasal cavity (*n* = 13; 7.97%), sphenoid (*n* = 11; 6.74%), middle turbinate (*n* = 7; 4.29%), frontal sinus (*n* = 4; 2.45%), and superior turbinate (*n* = 1; 0.61%).

### Symptoms

The most frequently reported symptom was nasal obstruction, in 119 cases (73.00%), followed by epistaxis (*n* = 111; 68.09%), facial swelling (*n* = 27; 16.56%), facial pain and/or headache (*n* = 20; 12.26%), nasal discharge (*n* = 13; 7.97%), orbital anomalies (*n* = 11; 6.74%), and hyposmia (*n* = 9; 5.52%).

**Fig. 3:**
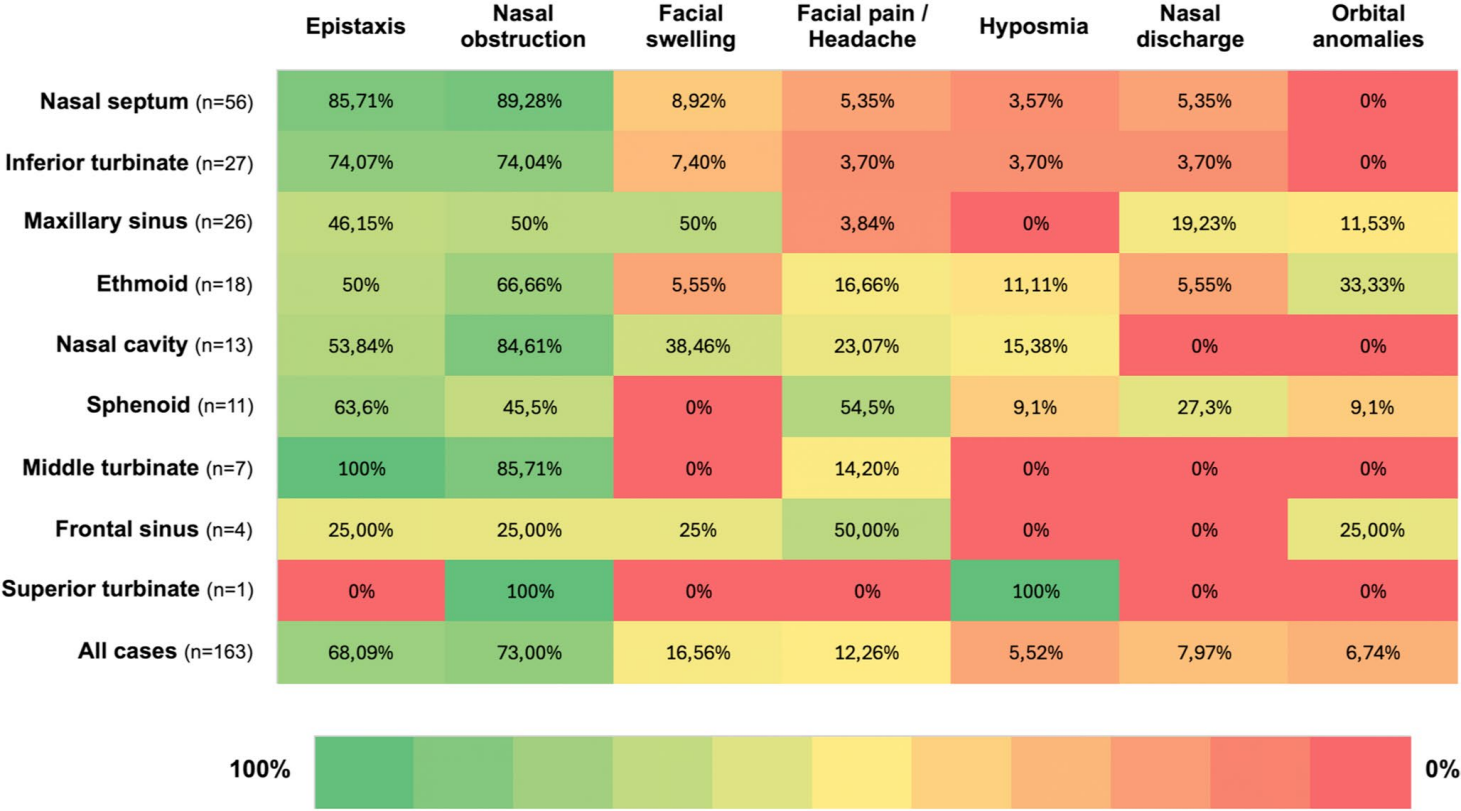
Frequency of the most reported symptoms according to the tumor site of origin

The combination of nasal obstruction and epistaxis was reported in 90 cases (55.21%), and this co-occurrence was more common when the tumor was located in the nasal septum (75.00%) and inferior turbinate (59.25%). Figure 3 illustrates the most reported symptoms for each tumor site.

Other symptoms were reported less frequently, such as snoring (*n* = 5) [6–10] rhinolalia (*n* = 3) [11], and sleep disturbance (*n* = 2) [12, 13]. Symptoms reported in only 1 case each included hematemesis [13], hyporexia [13], crusting [14], facial hypoesthesia [15], pus from the inferior canaliculus [6], difficulty eating [6], palatal swelling [16], loose teeth [17], pain when chewing [17], tumor erosion through the skin [18], hemoptysis [19], tumor in the nose [20], epiphora [21], asymptomatic (incidental) [22], pharyngeal discomfort with dysphagia [23], ipsilateral hearing loss [23], cough [24], sore throat [24], fever [24], trismus [24], impaired vision [25], marked thirst [25], and diplopia [26]. 

### Duration of symptom progression

In 20 cases, the duration of symptom progression was not reported. Four cases were described as “emergency,” [27–30] 2 as congenital [20–31], and 1 as an incidental finding [22]. One case exhibited a very slow progression, described as “more than 20 years.” [32].

For the remaining cases, the mean (SD) duration of symptom progression was 8.81 (15.82) months, with a median of 3 months.

### Imaging

The majority of patients underwent computed tomography (CT)— 40 cases did not involve CT, most of which occurred before the 1990s. Among the 60 cases where the contrast enhancement pattern of the tumors was described, 4 (6.66%) showed no contrast enhancement, 9 (15.00%) had mild contrast enhancement, 1 (1.66%) had moderate enhancement, 8 (13.33%) had heterogeneous enhancement, 28 (46.66%) showed homogeneous enhancement or were classified as an “enhancing tumor”, and 9 (15.00%) had intense or strong enhancement.

Magnetic resonance imaging (MRI) was performed in 30 cases (18.4%), all of which were described as solid lesions, except for 1 case described as a cystic lesion [33]. Of the 18 cases with a description of the contrast enhancement pattern, 9 (50.00%) reported intense contrast enhancement and 7 (38.88%) reported only “contrast enhancement.” Six cases described flow voids.

### Angiography and embolization

Twenty-nine patients (17.79%) underwent preoperative angiography, but the tumor-feeding vessels were not specified in 3 of these cases. Among the remaining 26 cases, 6 (23.07%) had negative results (no feeding vessels or no hypervascularity). In 15 cases (56.69%), the tumor was exclusively supplied by the arterial system of the internal maxillary artery (IMAX) and its terminal branches. In 2 cases (7.69%), the tumor was concomitantly supplied by the IMAX and facial artery. The tumor was supplied by the IMAX and ethmoidal branches of the ophthalmic artery in 2 cases (7.69%), and by the ophthalmic artery alone in 1 case (3.84%).

Embolization was performed in 19 patients (11.65%), with 18 procedures occurring preoperatively and 1 postoperatively due to bleeding [34]. 

### Preoperative biopsy

Preoperative biopsy was performed in 54 cases (33.12%). Of these, 24 (44.45%) showed brisk bleeding. In 20 cases (37.03%), the biopsy did not yield a diagnosis of angiofibroma, which was subsequently confirmed by the anatomopathologic examination of the surgical specimen.

### Immunohistochemistry

Immunohistochemistry was performed in 35 cases (21.47%), but detailed findings were unavailable for 5 of these cases. CD34 was the most positive marker (21/30), followed by alpha-actin (15/30), vimentin (10/30), beta-catenin (7/30), CD31 (7/30), androgen receptor (5/30), and CD117 (3/30). Factor VIII, factor XIIIa, LCA, and ERG were each reported as positive in only 1 case (1/30).

### Treatment and recurrence

Among the 163 cases, 1 patient refused treatment [35] and 1 experienced spontaneous autoamputation of the tumor without recurrence [36]. 

A combination of surgery and radium seeds was used in 3 cases [37, 38]. One patient received neoadjuvant radiotherapy followed by surgery [16]. None of these cases showed recurrence.

Radiotherapy was the initial treatment in 3 cases, but all of them exhibited persistent tumors; 2 patients underwent salvage surgery [38], and 1 died [25]. 

Of the 154 cases where surgery was the first-choice treatment, recurrence data were not reported in 21 cases. One case with incomplete resection underwent definitive surgery [18]. Seven patients experienced recurrence after definitive surgery, all of whom underwent revision surgery, with 1 patient also receiving radiotherapy for a persistent tumor [17]. 

The recurrence rate for cases where surgery was the initial approach was 5.26%, occurring at a mean (SD) of 3.75 (3.78) months (median, 2 months) after surgery. In these cases, the tumor originated from the maxillary sinus (*n* = 3), ethmoid (*n* = 2), and inferior turbinate (*n* = 2).

**Fig. 4:**
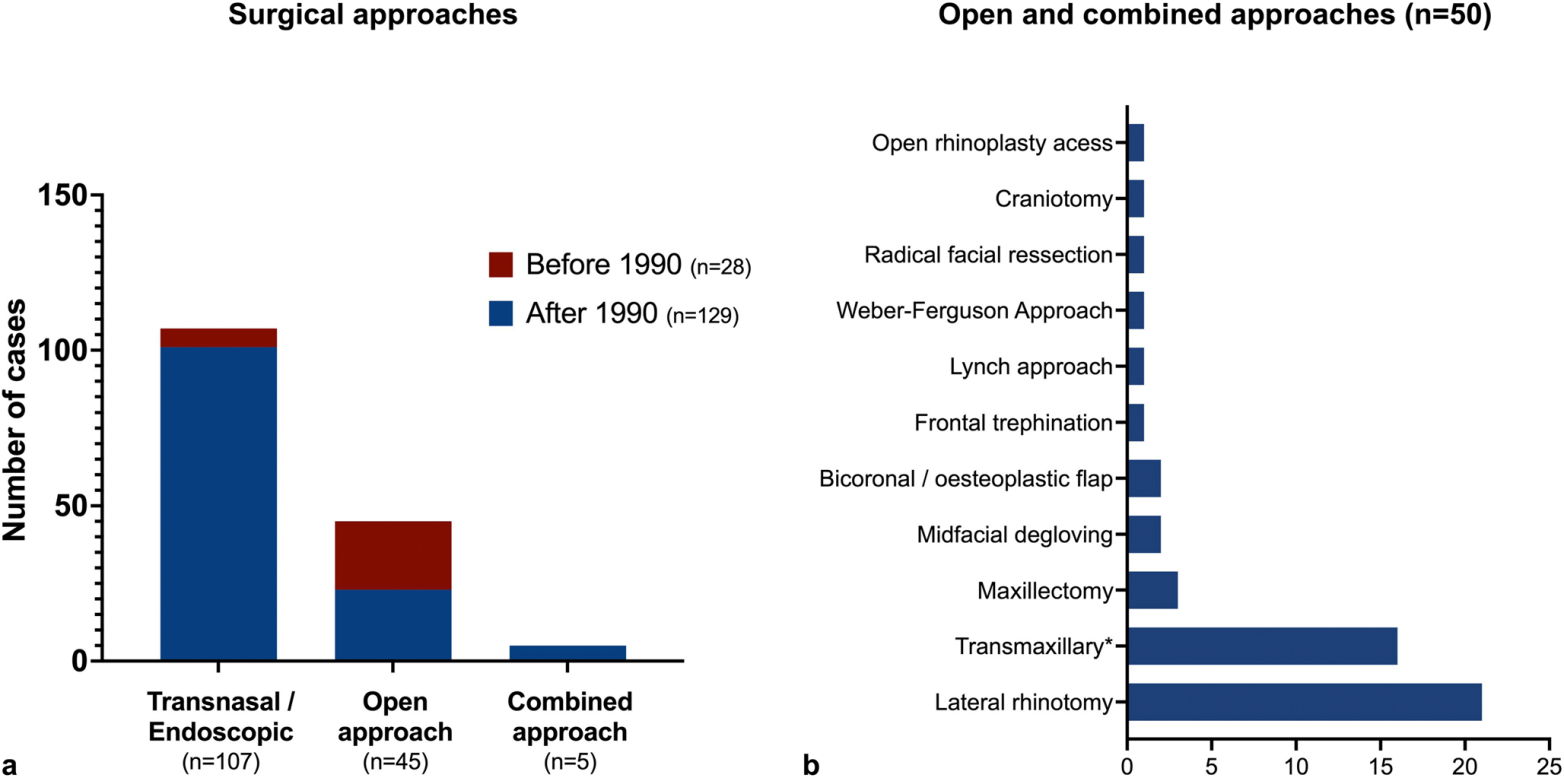
**A**, overview of the surgical approaches, comparing before and after 1990. **B**, open and combined approaches performed

Surgery was performed at some point during treatment (first choice or salvage) in 160 cases (98.15%). Transnasal resection (including endoscopic resection) was performed in 107 cases (66.87%), open surgery in 45 (28.12%), and combined (endoscopic and open) approaches in 5 (3.12%); the specific type of surgery was not reported in 3 cases (1.87%). Figure 4a provides a detailed overview of the surgical approaches, comparing their application before and after 1990. Figure 4b specifies the open approaches performed.

### Treatment complications

There was one case of soft palate necrosis after embolization [9]. In 1 case, radiotherapy treatment was discontinued due to bleeding [25]. The same patient subsequently developed meningitis after craniotomy and transnasal approaches for a lesion biopsy involving the central nervous system.

Of the 160 patients undergoing surgery, 21 (13.12%) experienced significant intraoperative bleeding. In 3 of these cases, the procedure was stopped, and a staged revision surgery was required [39–41]. Postoperative bleeding occurred in 4 cases (2.5%) [19, 34, 37, 42]. 

### Prognosis and mortality

Intracranial and orbital involvement were reported in 2 cases, both occurring in recurrent tumors [18, 43]. Intracranial involvement alone was reported in 1 case, which was the only case of patient death [25]. Involvement of the inferior orbital fissure occurred in 2 cases [44, 45]. 

There was 1 case of malignant transformation [38]. The patient had a primary tumor in the maxillary sinus, which was treated with radiotherapy without success, followed by surgery with radium tubes. Nine years later, a tumor developed in the middle turbinate, with a pathologic diagnosis of fibrosarcoma.

### Follow-up

Follow-up duration was not reported for 44 cases. For the remaining 119 cases, follow-up duration ranged from 1 to 132 months, with a mean (SD) duration of 19.81 (21.73) months, and a median of 12 months.

### Narrative summary

A detailed narrative summary of the included cases is presented in Table 1.

**Table 1:** Narrative summary of included cases

Author, Year of publication	Gender, age (years)	Side, site of origin	Symtoms	Duration (months)	Investigation	Pre-op biopsy: bleeding/ENA diagnosed	IH	Treatment	Follow up (months)
Gazmenga, 2025 [77]	M, 42	R, Nasal cavity/nasal septum	EP, NOB	12	CT + MRI	-	β-catenin, CD31, α-SMA	Endoscopic resection	12
Tawfik, 2025 [32]	M, 27	Bilateral, Frontal sinus	FS	“more than 20 years”	CT + MRI + Angio (“no definite feeding vessels”)	-	CD34, CD31, vimentin, α-SMA	Bicoronal approach	12
Grgurević, 2025 [78]	F, 26	L, Nasal septum (posterior)	EP, NOB	18	CT + Angio (IMAX)	-	CD34	Endoscopic resection + EMB	8
Basharat, 2025 [79]	M, 20	L, Nasal Septum (anterior)	EP, NOB, FS (Nasal)	0,3	CT	-	-	Transnasal resection	12
Yuen, 2025 [80]	M, 46	R, Nasal septum (CP)	EP, NOB, FS (Nasal)	4	CT	-	-	Transnasal resection	6
Adra, 2024 [81]	M, 37	R, Sphenoid Sinus (floor)	EP, NOB, FP/H, HYP, ND	12	CT + Biopsy	No/No	-	Endoscopic resection	NR
Hussain, 2024 [82]	F, 44	L, Middle Turbinate	EP, NOB	2	CT	-	-	Midfacial degloving	15
Valentini, 2023 [83]	M, 34	Bilateral, Frontal Sinus (Frontal Beak)	EP, NOB	9	CT + MRI + Biopsy	No/Yes	CD 31, CD34, α-SMA, β-catenin, vimentin, AR	Combined: endo + OPF approach	24
Cheah, 2023 [84]	M, 25	R, Maxillary Sinus	NOB, ND	26	CT + Biopsy	No/No	-	Endoscopic medial maxillectomy	3
Mahajan, 2023 [85]	M, 22	R, Maxillary Sinus (lateral wall)	NOB, FS (Nose swelling)	3	CT	-	-	Caldwell luc approach	3
Mustafa, 2022 [10]	M, 6	L, Nasal septum	EP, NOB, Snoring	2	CT	-	-	Endoscopic resection	12
Kurien, 2022 [26]	M, 22	R, Frontoetmoidal	FP/H (Headache), OA (Proptosis); Diplopia	1	CT + MRI + Angio (ophthalmic artery)	-	-	Endoscopic resection	24
Yan, 2022 [9]	M, 4	L, Inferior turbinate (Left, tail) and lateral wall nasopharynx	EP, NOB, ND (Purulent discharg), Snoring	0,3	CT + MRI + Angio (IMAX)	-	-	Endoscopic resection + EMB (2x). Soft palate necrosis after BEM	3
Wan, 2022 [15]	M, 14	R, Maxillary Sinus (Ant wall)	EP, NOB, FS, OA (hypertelorism), facial hypoaesthesia	2	CT + MRI + Biopsy	No/Yes	CD34	Hemifacial degloving	6
Yeon, 2022 [86]	M, 27	R, Ethmoid - Nat Ostium Maxillary Sinus (lat. Wall)	NOB	4	CT	-	-	Endoscopic resection	13
Hassan, 2022 [87]	M, 13	L, Middle Turbinate	EP, NOB	3	CT	-	β-catenin, AR	Endoscopic resection	4
Thobejane, 2021[88]	M, 23	R, Nasal septum	EP	12	CT	-	-	Endoscopic resection	NR
Uwents, 2021[89]	F, 6	L, Ethmoid	NOB, OA (proptosis)	NR (several months)	CT + MRI + Angio (SPA branches) + Biopsy	Yes/No	ERG, CD34, CD31, AR, α-SMA	Endoscopic resection + EMB	6
Janaú, 2020 [23]	M, 12	R, Sphenoid Sinus	EP, NOB, pharyngeal discomfort, dysphagia, ipsilateral hearing loss	12	CT	-	-	Endoscopic resection/Bleeding (BT)	12
Shamim, 2020 [90]	M, 38	R, Nasal septum	EP, NOB	NR	CT	-	-	Lateral rhinotomy	NR
Law, 2020 [91]	F, 51	L, Nasal Septum (anterior)	EP, NOB, FS (Nasal swelling)	2	CT + MRI + Angio (not described) + Biopsy	No/No	-	Endoscopic resection + EMB	24
Sundaram, 2019 [18]	M, 32	L, Ethmoid	FP/H (headache and eye pain), OA (Proptosis), erosion of the tumor through the skin.	NR	CT + MRI + PET-CT (hypermetabolic, SUV-max 6.8)	-	CD117, AR	Combined: endo + bifrontal craniotomy and duraplasty. Previously treated with 3 transnasal-transorbital surgeries.	NR
Castillo, 2019 [92]	M, 32	L, Nasal septum (CP)	EP, NOB	NR	CT + Angio (IMAX, infraorbital, SPA; septal Branch of AEA) + Biopsy	Yes/Yes	-	Endoscopic resection	12
Shobana, 2019 [93]	M, 15	R, Nasal septum	EP, NOB	2	MRI	-	-	Transnasal resection	NR
Sobhari, 2019 [94]	M, 25	L, Inferior turbinate (inferoanterior)	EP, NOB	6	CT	-	-	Endoscopic resection (bleeding)	NR
Kujundžić, 2019 [95]	F, 35	L Nasal Septum (anterior)	EP, NOB, FP/H (Facial pain)	12	CT + MRI + Angio (SPA branches)	-	CD-34, α-actin	Endoscopic resection + EMB	48
Spinosi, 2019 [96]	M, 28	R, Nasal Septum (anterior)	EP, NOB	Emergency (0,5)	CT	-	CD-34	Endoscopic resection	6
Brown, 2019 [97]	M, 49	L, Inferior turbinate (anteromedial)	EP, NOB	5	CT + Angio (IMAX) + Biopsy	No/Yes	-	Endoscopic resection + EMB	NR
Kim, 2019 [98]	M, 18	R, Frontal sinus (lateral wall)	FP/H (Facial pain)	0,25	CT	-	-	Combined: endo + frontal trephination (incomplete resection, followed by subsequent tumor regression)	60
Ghazizadeh, 2018 [99]	M, 40	L, Sphenoid Sinus (anterolateral wall)	EP, NOB, Headache	2	CT + MRI + Biopsy	No/Yes	-	Endoscopic resection	24
Gupta, 2018 [100]	M, 13	R, Nasal Septum (posterior, BP)	EP	Emergency (3 days)	CT	-	-	Endoscopic resection	6
Lee, 2018 [101]	M, 43	R, Nasal Septum (BC Junction)	NOB	0,5	CT	-	AR	Endoscopic resection	12
Kim, 2018 (case 1) [102]	M, 41	R, Middle Turbinate (posterior end)	EP	NR	CT	-	-	Endoscopic resection	19,2
Kim, 2018 (case 2) [102]	M, 22	Inferior turbinate	NOB	NR	CT	-	-	Endoscopic resection	15
Tyagi, 2018 [103]	F, 54	L, Nasal Septum (Antero-superior; bony septum)	EP, NOB	12	CT	-	-	Endoscopic resection	NR
Türk, 2018 [104]	M, 43	L Nasal Cavity (vestibule)	NOB, FS (nasal tip)	8	CT + MRI + PET-CT (SUV-max 4.3) + Biopsy	No/No	CD-34	Resection (not specified)	NR
Singh, 2018 [105]	F, 9	L, Nasal Septum (anterior)	EP, NOB	3	CT	-	CD34, α-SMA, vimentin	Endoscopic resection	12
Toplu, 2018 [106]	M, 13	L, Middle Turbinate (anteroinferior part)	EP, NOB	3	CT	-	CD-34	Endoscopic resection	1
Pandey, 2017 [107]	F, 40	R, Nasal Cavity - (lateral wall -uncinate process)	EP, NOB, FP/H, HYP, FS (right side alar crease)	120	CT + Biopsy	Yes/No	-	Lateral rhinotomy (bleeding)	NR
Jaramillo, 2017 [13]	F, 5	R, Nasal Septum (anterior)	EP, hematemesis, hyporexia, obstructive breathing symptoms and interrupted sleep	2	CT + MRI + Angio (IMAX)	-	-	Endoscopic resection + EMB	12
Althbety, 2017 [43]	M, 30	L, Ethmoid	NOB, HYP, OA (Proptosis), FP/H (Headache)	3	CT + MRI + Angio (“No feeding wessels”) + Biopsy	No/Yes	-	Combined: endo + Lynch; 8 months after recurrence (orbit and intracranial); 2nd surgery (endoscopic)	9
Mahdah, 2017 [108]	M, 21	L, Maxillary Sinus	FS (cheek)	4	CT + MRI + Angio (IMAX + Facial arteries) + Biopsy	No/Yes	-	Weber-Ferguson approach + EMB	6
Ganguly, 2017 [109]	M, 7	L, Nasal septum (antero-inferior)	EP, NOB	2	CT	-	-	Endoscopic resection	12
Hwang, 2016 [39]	F, 59	R, Nasal Cavity (lateral Wall)	EP, NOB	12	CT + MRI + ANGIO (SPA)	-	-	Endoscopic medial maxillectomy + EMB (1st attempt interrupted by bleeding; 2nd post-EMB. Underwent RT + immunotx for concurrent melanoma)	12
Mahmood, 2016 [110]	M, 49	L, Nasal Cavity (Vestibule/lateral nasal wall)	EP, NOB, HYP, FP/H (Headaches)	1	CT + MRI + Angio (IMAX + Facial arteries)	-	Not specified	Transnasal resection + BEM	6
Azevedo, 2016 [111]	F, 12	L, Maxillary Sinus (posterosuperior wall)	EP, NOB	6	CT	-	Not specified	Combined: endo + Caldwell-Luc	84
Singh, 2016 [112]	F, 28	R, Inferior turbinate	EP, NOB	6	CT	-	CD34, α-SMA, vimentin	Endoscopic resection	12
Mutlu, 2015 [113]	M, 48	R, Inferior turbinate (tail)	NOB	12	CT	-	Not specified	Endoscopic resection	6
Al Osaimi, 2015 [12]	M, 14	R, Nasal Septum (anterior, CP)	EP, NOB, HYP, FP/H, ND (Post nasal drip), sleep disturbance	3	CT	-	-	Transnasal resection	NR
Nazar, 2015 [114]	M, 9	L, Inferior turbinate (head)	EP	NR	CT	-	-	Endoscopic resection	12
Debbarma, 2015 [115]	F, 12	L, Inferior turbinate	EP, NOB	6	CT	-	-	Endoscopic resection	12
Ewe, 2015 [35]	F, 67	R, Nasal Septum (antero-superior)*	NOB	6	CT + Biopsy	No/Yes	-	Refused treatment; mass remained	12
Salimov, 2015 [116]	F, 26	R, Inferior turbinate (posterior)	EP	2	MRI	-	-	Endoscopic resection + EMB	13
Al Khatib, 2015 [117]	M, 13	L, Nasal Septum (BC Junction)	EP, NOB, FS (Nasal)	5	CT	-	-	Endoscopic resection	18
Niedzielski, 2015 [118]	F, 9	L, Nasal Septum (anterior, CP)	EP	Emergency	CT	-	CD34	Transnasal resection	NR
Ismi, 2015 [30]	M, 22	R, Maxillary Sinus	EP	Emergency	No imaging	-	CD 31	Endoscopic resection	12
Shanmugam, 2014 [119]	M, 13	R, Nasal Septum (anterior, CP)	EP, NOB	NR	CT	-	-	Endoscopic resection	NR
Singhal, 2014 (Case 1) [120]	M, 28	L, Ethmoid	EP, NOB	9	CT	-	-	Endoscopic resection (bleeding)	6
Singhal, 2014 (Case 2) [120]	M, 12	L, Nasal Septum (anterior, CP)	EP, NOB	1,5	CT	-	-	Transnasal resection	NR
Karthikeyan, 2014 [121]	M, 13	L, Sphenoid Sinus (floor, left)	EP	12	CT + Angio (IMAX)	-	-	Endoscopic resection + EMB	6
Son, 2014 [122]	M, 68	L, Superior Turbinate	NOB, HYP	84	CT	-	CD34, α-SMA, β-catenin	Endoscopic resection	12
Baptista, 2014 [8]	F, 8	R, Inferior turbinate (não especificado)	EP, NOB, HYP, snoring.	6	CT	-	Not specified	Endoscopic resection (bleeding)	6
Rao, 2014 [123]	F, 27	Bilateral, Ethmoid	OA (Telechantus and proptosis)	NR	MRI	-	-	Craniofacial ressection, via lateral rhinotomy (Previously operated; recurrence occurred)	18
Keskin, 2013 [40]	M, 21	R, Sphenoid Sinus (lower lateral wall)	FP/H (Headache)	12	CT + MRI + Angio-MRI + Angio (IMAX; 2nd approach: feeding from the cavernous segment of ICA)	-	-	Endoscopic resection + EMB (1st attempt interrupted by bleeding; reop 6 months later)	18
Szymańska, 2013 (Case 1) [11]	M, 31	L, Nasal septum	NOB, rhinolalia	3	X-ray	-	-	Transnasal resection	NR
Szymańska, 2013 (Case 2) [11]	M, 42	R, Nasal septum	EP, NOB, rhinolalia	4	X-ray	-	-	Transnasal resection	NR
Szymańska, 2013 (Case 3) [11]	F, 18	L, Nasal septum	EP, NOB, rhinolalia	2	X-ray	-	-	Transnasal resection	NR
Szymańska, 2013 (Case 4) [11]	F, 40	R, Nasal septum	EP, NOB, FP/H, ND (mucopurulent)	12	CT	-	-	Transnasal resection	NR
Szymańska, 2013 (Case 5) [11]	M, 12	R, Nasal septum	EP, NOB	1	CT	-	-	Transnasal resection	NR
Szymańska, 2013 (Case 6) [11]	M, 15	R, Nasal septum	EP, NOB	3	CT	-	-	Transnasal resection	NR
Szymańska, 2013 (Case 7) [11]	F, 47	R, Nasal septum	NOB	4	CT	-	-	Transnasal resection	NR
Maheshwarappa, 2013 [124]	M, 19	L, Inferior turbinate (anteroinferior)	EP, NOB	4	CT	-	-	Transnasal resection (bleeding)	NR
Correia, 2013 [7]	NR, 10	R, Nasal septum	NOB, HYP, Snoring	6	CT	-	-	Endoscopic resection	21
Kang, 2013 [125]	M, 16	L, Inferior turbinate (anterior)	NOB	2	CT + Biopsy	No/Yes	-	Endoscopic resection (recurred in 2 months; new resection)	12
Lee, 2013 [126]	F, 52	L, Inferior turbinate (anterior)	EP, NOB	3	CT + MRI	-	-	Endoscopic resection	NR
Atmaca, 2013 [127]	M, 37	R, Nasal septum (posterior)	EP, NOB	48	CT	-	-	Endoscopic resection	7
Zulkifli, 2013 [128]	M, 28	R, Inferior turbinate (posterior)	EP, NOB	3	CT + Biopsy	Yes/No	-	Endoscopic resection	10
Peric, 2013 [69]	F, 63	L, Middle Turbinate (anteroinferior)	EP, NOB, FP/H	8	CT + Biopsy	Yes/Yes	CD34, CD31, α-SMA, vimentin, Factor VIII	Endoscopic resection	60
Malipatil, 2013 [129]	M, 60	L, Nasal Cavity (Vestibule, lateral aspect)	NOB	12	CT	-	-	Transnasal resection	6
Mahajan, 2013 [130]	F, 50	R, Nasal septum (CP, Anterosuperior)	EP, NOB	6	CT	-	-	Endoscopic resection	12
Anand, 2013 [131]	F, 40	R, Nasal Septum (CP, anteroinferior)	EP, NOB	4	CT	-	-	Transnasal resection	4
Narve, 2013 [29]	F, 40	R, Nasal Septum (anterior, CP)	EP, NOB, ND	2	CT	-	-	Transnasal resection	2
Dogan, 2013 [66]	M, 16	L, Nasal septum (caudal)	NOB	48	CT	-	CD34, α-SMA, β-catenin	Transnasal resection	6
San, 2012 [33	F, 49	R, Sphenoid sinus	EP, FP/H (Headache)	1,5	CT + MRI	-	-	Endoscopic resection	10
Hod, 2012 [132]	F, 56	R, Inferior turbinate	FS (nasal vestibule)	36	CT	-	-	Transnasal resection	2
Malvic, 2012 [133]	M, 14	L, Maxillary sinus (lateral wall)	NOB, ND (Rhinorrea)	NR	CT	-	-	Endoscopic resection (bleeding)	3
Berkiten, 2012 [134]	F, 26	R, Nasal septum	EP, NOB	4	Biopsy	No/No	-	Transnasal resection	NR
Garcia-Rodriguez, 2012 (Case 1) [135]	M, 60	R, Nasal Septum (anterior)	EP	2	CT	-	CD34 α-SMA, β-catenin, CD117	Transnasal resection	24
Garcia-Rodriguez, 2012 (Case 2) [135]	M, 56	R, Nasal Septum (anterior)	NOB	NR	CT + MRI	-	CD34 α-SMA, β-catenin, CD117	Endoscopic resection	27
Chavan, 2012 [136]	F, 21	R, Nasal Septum (anterior, CP)	EP, NOB	NR	CT	-	Not specified	Endoscopic resection	NR
Lee, 2012 [14]	F, 57	R, Inferior turbinate	FP/H (Nasal pain), crusting	2	CT	-	CD34, α-SMA	Endoscopic resection	3
Lerra, 2012 [137]	F, 16	L, Ethmoid (	EP, NOB, OA (telechantus), FS (Nasal dorsum)	24	CT + Biopsy	Yes/Yes	-	Lateral rhinotomy	NR
Hamdan, 2012 [27]	M, 19	R, Nasal septum (BC junction)	EP, NOB	“Weeks/emergency”	CT	-	-	Lateral rhinotomy	NR
Madana, 2012 [67]	F, 37	R, Nasal Septum (CP, anteroinferior)	EP, NOB	4	CT	-	CD34, α-SMA, vimentin	Endoscopic resection	22
Martinez, 2011 [138]	M, 54	R, Nasal septum (BP)	EP	NR (long history)	CT + MRI	-	-	Lateral rhinotomy	36
Bhagat, 2011 [139]	M, 28	R, Maxillary sinus (lateral wall)	FS (Cheek), OA (Proptosis), FP	1	CT + Biopsy	Yes/No	-	Lateral rhinotomy	2
Singh, 2010 [140]	M, 18	L, Nasal Septum (anterior, CP)	EP, NOB	3	CT	-	-	Endoscopic resection	12
Mohindra, 2009 [141]	M, 22	L, Nasal septum (CP, antero-inferior)	EP, NOB, FS (Nasal)	2	CT	-	-	Transnasal resection	24
Lin, 2009 [142]	M, 66	NR, Sphenoid Sinus	EP, NOB	NR	CT + Angio (not described)	-	-	Lateral rhinotomy + EMB	30
Uyar, 2009 [143]	M, 19	L, Nasal septum (BC junction)	EP, NOB	6	CT	-	CD31, Vimentin, Factor XIIIa	Transnasal resection	5
Zhou, 2008 [144]	M, 40	R, Inferior turbinate (posterior)	EP, NOB	0,67	CT + Angio (not described)	-	α-SMA	Endoscopic resection	18
Tasca, 2008 [145]	F, 57	R, Nasal septum (posterior)	NOB	12	CT	-	-	Transnasal resection	12
Durko, 2007 [146]	F, 26	L, Nasal cavity (extensio to anterior ethmoid)	NOB	2	CT + Biopsy	No/No	α-SMA, vimentin, LCA	Lateral rhinotomy	60
Cho, 2007 [147]	M, 72	L, Ethmoid	EP, NOB, HYP	6	CT + Angio (no hipervascularity) + Biopsy	Yes/No	-	Transnasal resection	36
Castillo, 2006 [36]	M, 9	L, Nasal Septum (anterior)	EP, NOB	NR (several month)	CT + MRI	-	-	Spontaneous resolution (tumor autoamputation)	9
Nomura, 2006 [148]	M, 62	L, Inferior turbinate (anterior)	EP, NOB	2	CT + MRI + Angio (SPA) + Biopsy	No/Yes	CD34	Transnasal resection	10
Lim, 2005 [149]	F, 41	L, Middle turbinate (medial face)	EP, NOB	2	CT + MRI + Angio (SPA)	-	-	Endoscopic resection + EMB	6
PARK, 2005 [28]	M, 57	L, Inferior turbinate (anterior)	EP	Emergency	CT	-	-	Endoscopic resection	6
Windfuhr, 2004 [150]	F, 13	L, Maxillary sinus	FS	2	CT + Angio (no hipervascularity) + Biopsy	No/Yes	-	Lateral rhinotomy	NR
Celik, 2004 (Case 1) [151]	M, 33	L, Inferior turbinate (anterior)	EP, NOB, FS (Nasal)	3	CT + Biopsy	No/Yes	-	Lateral rhinotomy with medial maxillectomy	NR
Celik, 2004 (Case 2) [151]	M, 60	R, Inferior turbinate (tail)	NOB	12	No imaging	-	-	Endoscopic resection	NR
Garcia, 2004 [52]	F, 60	R, Inferior turbinate (posterior)	EP, NOB	3	CT + MRI + Angio (IMAX)	-	-	Endoscopic resection	NR
Taggarshe, 2004 [34]	M, 30	R, Inferior turbinate (posterior)	EP, NOB	6	No imaging (emergency case)	-	-	Endoscopic resection - post-op EMB (bleeding)	14
Panesar, 2004 [6]	M, 1	L, Maxillary sinus (anterior wall)	FS, pus inferior canaliculus, snoring, difficulty feeding	1	CT + US	-	CD34, vimentin	First transnasal (heavy bleeding). Mass persisted (incomplete resection?). Midfacial degloving performed.	NR
Akbas, 2003 (Case 1) [152]	F, 50	R, Nasal septum (antero-inferior)	EP, NOB	24	CT	-	-	Transnasal resection	75
Akbas, 2003 (Case 2) [152]	F, 34	L, Nasal Septum (anterior)	EP, NOB	12	CT	-	-	Transnasal resection	65
Crespo Del Hierro, 2002 [153]	M, 17	R, Nasal Cavity (lateral wall)	EP, NOB	3	CT + Angio (SPA) + Biopsy	No/Yes	-	Endoscopic resection + EMB	24
Handa, 2001 [41]	M, 8	L, Nasal septum (BC junction)	EP, NOB	6	CT	-	-	Endoscopic attempt aborted due to heavy bleeding. Second via left alar crease incision	6
Mehta, 2001 [19]	M, 39	L, Ethmoid (anterior)	haemoptysis	3	CT + X-ray + Biopsy	No/No	-	Lateral rhinotomy (bleeding)	3
Shah, 2000 [154]	M, 32	R, Nasal septum (BC junction)	EP, NOB	2	CT + X-ray	-	-	Transnasal resection (bleeding)	6
Huang, 2000 [155]	M, 14	R, Middle turbinate (medial surface)	EP, NOB	3	CT + Angio (SPA + Ophtalmic) + Biopsy	Yes/No	-	Lateral rhinotomy with partial medial maxillectomy + EMB	12
Schick, 1997 (Case 1) [22]	M, 1	L, Nasal Cavity (lateral wall)	FS (medial canthus)	0,5	CT	-	vimentin	Transnasal resection	18
Schick, 1997 (Case 2) [22]	M, 9	R, Nasal Cavity (lateral wall/maxilla bone)	EP, FS	12	CT + Biopsy	No/Yes	-	Transnasal resection	12
Schick, 1997 (Case 3) [22]	M, 5	R, Sphenoid Sinus	Accidental (diagnosis during adenoidectomy)	Accidental	CT + Biopsy	No/Yes	-	Transnasal resection	72
Tsunoda, 1998 [24]	M, 12	L, Sphenoid Sinus	ND, FP/H (Cheek), cough, sore throat, fever and trismus	Emergencial (2 days)	CT + MRI + Angio (SPA) + Biopsy	Yes/Yes	-	Combined: endo + transmaxillary (+ EMB)	30
Gaffney, 1997 [156]	M, 9	L, Inferior turbinate (anterior)	EP	4	CT + Biopsy	Yes/Yes	-	Lateral rhinotomy	36
Alvi, 1996 [157]	F, 78	R, Inferior turbinate (anterior)	EP, NOB	2	CT + Biopsy	Yes/No	-	Transnasal resection	36
Hersh, 1995 [158]	F, 69	L, Nasal cavity (valvut area/vestibule)	NOB, FP/H (nasal disfomfort)	3	Biopsy	No/Yes	-	Open rhinoplasty approach	NR
Bhargava, 1995 [159]	M, 24	R, Nasal Septum (Midpart, CP)	EP, NOB	6	CT + X-ray	-	-	Transnasal resection	NR
Pastor Quirante, 1994 [42]	M, 71	R, Inferior turbinate	EP	3	CT	-	-	Transnasal resection - recurrence, reop (transnasal) 1 month after/Bleeding (BT)	12
Kitano, 1992 [16]	M, 13	L, Maxillary sinus	Palatal swelling	NR	CT + Scintigram + Biopsy	Yes/Yes	-	RT (20 Gy), followed by partial maxillectomy	36
Manjalay, 1992 [31]	M, Congenital	R, Maxilary sinus/maxilla (ant. wall)	FS	Congenital	CT + Biopsy	Yes/No	-	Sublabial incision, carbon dioxide laser used	36
Sarpa, 1989 [160]	M, 9	R, Nasal septum (BC junction)	EP, NOB	1,5	CT + X-ray + Angio (negative) + Biopsy	No/Yes	-	Transnasal resection	6
Nasretdinov, 1985 [161]	F, 33	L, Nasal cavity (lateral wall)	EP, NOB	3	Biopsy	No/Yes	-	Transmaxillary ressection (Denker approach), wih LECA	6
Sakurai, 1984 (Case 1) [162]	M, 37	L, Nasal septum	EP, NOB	2	CT + X-ray + Biopsy	No/No	-	Transnasal resection	36
Sakurai, 1984 (Case 2) [162]	F, 37	L, Ethmoid (anterior)	EP, NOB	2	X-ray	-	-	Surgery (not specified)	24
Hiraide, 1984 [163]	M, 13	L, Nasal Septum (anterior)	EP, NOB	2	X-ray + Biopsy	Yes/Yes	-	Transnasal resection	60
Obiako, 1983 [164]	M, 12	R, Nasal cavity (lateral wall)	NOB, FS (nasal)	4	X-ray + Biopsy	Yes/No	-	Lateral rhinotomy (diethylstibestrol preop)/Bleeding (BT)	NR
Kim, 1982 [57]	F, 26	L, Inferior turbinate (anterior)	EP, NOB	5	X-ray	-	-	Transnasal resection	5
Juul, 1982 [165]	F, 27	L, Maxillary sinus (posterior and lateral walls)	NOB, FP/H (Headache), ND (purulent)	2	X-ray	-	-	Caldwell luc approach; recurrence with 2nd surgery 1 year after	NR (more than 1,5 year)
Tani, 1977 [166]	M, 27	R, Maxillary Sinus	EP	NR	X-ray	-	-	Caldwell luc approach	12
Ramanjaneyulu, 1974 [44]	M, 17	R, Maxillary sinus (posteromedial wall)	NOB, FS (Cheek)	6	X-ray + Biopsy	Yes/No	-	Lateral rhinotomy (bleeding/BT)	NR
Yamagiwa, 1974 [167]	M, 14	L, Sphenoid sinus	EP, NOB, FP/H (headache), ND (antero-posterior mucopurulent)	1	CT + X-ray + Biopsy	Yes/No	-	Transnasal resection (bleeding)	7
Ryc, 1973 [168]	M, 17	R, Maxillary Sinus (medial lower wall)	EP	3	CT + X-ray + Biopsy	Yes/Yes	-	Caldwell luc approach	12
Chakrabarti, 1973 [45]	M, 17	R, Maxillary sinus (posteromedial wall)	EP, NOB, FS	12	X-ray	-	-	Lateral rhinotomy (bleeding/BT)	NR
Pathak, 1970 [169]	M, 18	Maxillary sinus	EP, NOB, ND (postnasal drip)	3	X-ray + Biopsy	Yes/Yes	-	Denker’s approach; ligation ECA and EAE	94
Perko, 1969 [17]	F, 33	Maxillary sinus	Loose teeth, pain on mastigation	NR	X-ray	-	-	Transmaxillary resection; recurrence (2 months), resection of tumor and maxilla/zigoma); recurrence (3 months), maxillectomy; recurrence, RT.	NR (multiple recurrences)
Maniglia, 1969 [170]	M, 15	Maxillary sinus (POSTERIOR AND MEDIAL WALL)	EP, NOB, ND (purulent)	24	X-ray + Biopsy	Yes/Yes	-	Transmaxilarry ressection, weber-ferguson (criosurgery)	16
Szczepanski, 1967 [171]	M, 13	Ethmoid	EP	3	Biopsy	No/Yes	-	Transnasal resection	NR
Hiranandani, 1967 [172]	F, 30	Ethmoid (posterior)	EP, NOB, OA (Proptosis)	48	X-ray	-	-	Lateral rhinotomy, transpalatal incisions, LECA/Bleeding (BT)	NR
Ogura, 1965 [173]	M, 16	Maxillary Sinus	EP, FS	NR	X-ray + Biopsy	No/No	-	Transmaxillary (bleeding)	6
Reddy, 1963 [25]	F, 50	Sphenoid sinus	OA (Proptosis), Impaired vision, marked thirst	2	X-ray + Biopsy	No/Yes	-	RT. Initial craniotomy (extradural lesion in sphenoid) and transseptal biopsy. Developed meningitis and bizarre behavior. RT indicated. Death 18mo after (autopsy: tumor invaded pituitary, optic nerves, and nasopharynx).	18
Furstenberg, 1963 [20]	M, Congenital	Ethmoid (Anterior)	Tumor in the nose	Congenital (diagnosis at 5 weeks)	Phisical exam	-	-	Lateral rhinotomy	NR
Hora, 1962 [174]	M, 13	Maxillary sinus and ethmoid	EP, NOB	3	X-ray + Parotid sialogram	-	-	Transnasal + Caldwell Luc (bleeding, BT)	9
Alajmo, 1961 (Case 1) [38]	M, 58	Maxillary sinus/Ethmoid	FS	24	X-ray	-	-	Radical facial ressection + LECA + radium seeds for 72 h	84
Alajmo, 1961 (Case 2) [38]	M, 18	Ethmoid	EP, NOB	3	X-ray + Biopsy	No/Yes	-	Lateral rhinotomy + radium seeds 36 h	42
Alajmo, 1961 (Case 3) [38]	F, 26	Ethmoid	EP, FP/H (frontal)	60	X-ray + Biopsy	Yes/Yes	-	Lateral rhinotomy (3 years prior: endonasal polyp removal + RT)	36
Alajmo, 1961 (Case 4) [38]	M, 14	Ethmoid	NOB	4	X-ray + Biopsy	No/Yes	-	Lateral rhinotomy. Initially, family refused surgery; RT given (15 Gy). Recurrence; surgery performed 2 years later.	5
Alajmo, 1961 (Case 5) [38]	M, 6	Maxillary sinus (posterior wall)	NOB	12	X-ray + Biopsy	Yes/Yes	-	Biopsy via Caldwell-Luc with bleeding; tumor not removed. RT done twice (1st interrupted due to bleeding). Later, resection via lateral rhinotomy + radium tubes (24 h). After 9 yrs, malignancy (fibrosarcoma in middle turbinate): resection + radium tubes (cured)	132
Radcliffe, 1951 [21]	M, 16	Ethmoid	NOB, ND, Epiphora	14	X-ray	-	-	Transnasal/Transmaxillary resection (Caldwell luc) (bleeding)	NR (Several weeks)
Munson, 1941 [37]	M, 15	Maxillary Sinus	EP, FS, OA (Proptosis)	60	X-ray + Biopsy	Yes/Yes	-	Persistent bleeding after transmaxillary biopsy: bilateral LECA, left LCCA, radium capsule, BT. Transmaxillary approach after.	10
Tsuru, 1937 [175]	F, 33	Maxillary Sinus	EP, NOB, FS	4	-	-	-	Denker’s approach	NR
de Kleyn, 1918 [56]	F, 30	Ethmoid	EP, NOB	NR	X-ray	-	-	Denker’s approach	36

## Discussion

### Etiopathogenesis

Juvenile nasopharyngeal angiofibroma is acknowledged as a vascular malformation resulting from an incomplete regression of the first branchial arch artery - a so-called atavism. The artery exists only temporarily between day 22 and day 24 and recedes via a vascular plexus formation surrounding the maxillary/sphenopalatine artery. Embryological vascular cells are therefore found histologically in the region of the sphenopalatine fossa but may also be identified in other regions, whenever regression of vascular components was incomplete. The histological findings of vessels with only endothelial lining and incomplete vascular walls in a fibrous stroma containing inflammatory cells were supplemented by electronmicroscopic analyses and positive results in immunohistology, suggesting the embryologic origin (laminin α2; TSHZ1; collagen Iα1; collagen Iα2; collagen VI). Other reports support the idea that tumor formation is based on a disturbed epithelial to mesenchymal and vice versa transition due to an aberrant Wnt-signaling pathway. Moreover, receptors for androgen, estrogen, follicle-stimulating hormone, and luteinizing hormone were found, indicating the complex origin of these tumor-like lesions, predominantly in male adolescents but also in female patients and patients of any age [46]. Nevertheless, the etiopathogenesis of extranasopharyngeal angiofibromas arising in various locations of the sinonasal tract remains unclear.

#### Epidemiology

Reports of ENA-SNT have increased substantially in recent decades. While there are also reports suggesting a rise in the incidence of JNA cases [47, 48], it remains unclear whether the increased reporting of ENAs is due to a real increase in incidence or greater knowledge and awareness of the disease leading to more publications.

Although JNA is believed to have a higher incidence in India and Middle Eastern countries [48], robust epidemiological evidence is lacking, given the rarity of the disease. In contrast, this association appears to be true for ENA-SNT, with these countries accounting for 38.65% of reported cases and the Asian continent for 56.44%.

The term ‘juvenile’ in JNA refers to its striking epidemiological characteristic of occurring predominantly in male adolescents, with fewer occurrences in other age groups [49, 50] and exceptional rarity in females [51–54]. ENA-SNT also shows a preference for males, but with a more balanced male-to-female ratio of approximately 2:1. ENA-SNT can affect individuals across a wide age range, from 0 to 78 years, with a mean age of 28.8 years. There are also reports of 2 congenital cases [20, 31] and 3 cases in pregnant women [55–57]. 

### Clinical manifestations

The most common symptoms of JNA are unilateral nasal obstruction (75%−91%) and epistaxis (63%−68%) [58, 59], with other symptoms varying depending on the tumor progression pathway. Its occurrence in the pterygopalatine fossa facilitates spread through the foramina and fissures in the region.

Similar to JNA, the most common symptoms of ENA-SNT are also nasal obstruction (73.00%) and epistaxis (68.09%), frequently occurring in combination (55.21%). Other symptoms depend on tumor site. Facial swelling is more common in the maxillary sinus, while facial pain and orbital anomalies are more common in the frontal and ethmoid sinuses. This symptom frequency differs from that reported in a previous review by Windfuhr & Vent [2], since they included ENAs originating from other areas of the head and neck.

The mean time from the onset of symptoms to the diagnosis of ENA-SNT was 8.81 months, whereas for JNA, it ranges from 6 months to 2 years [58, 60, 61]. This diagnostic delay can occur because the symptoms are nonspecific and common to other sinonasal diseases.

Both JNA and ENA-SNT can exhibit expansive growth, remodeling, and invading adjacent structures. However, JNA’s more indolent growth in a more complex location contributes to its greater aggressiveness. In 40% of cases, diagnosis occurs at advanced stages, with intracranial extension occurring in up to 17% [62]. While there is no formal staging for ENAs, most cases compiled in this review did not show significant aggressiveness, with intracranial involvement in only 1.84% and orbital involvement in 2.44%.

### Investigation and diagnosis

The diagnosis of angiofibromas is based on clinical suspicion, nasal endoscopy, and imaging studies (MRI and CT), with confirmation by anatomopathologic examination. Preoperative biopsy is not indicated due to the risk of significant bleeding. In our sample, almost half of the cases (44.45%) showed brisk bleeding, and in almost one-third of the cases, the preoperative biopsy did not yield a diagnosis of angiofibroma, likely due to the bleeding and superficial samples [2]. 

While JNA typically presents intense and homogeneous contrast enhancement on imaging [63, 64], ENA-SNT may exhibit less intense and inhomogeneous enhancement. Our findings showed that only 61.66% of cases had a homogeneous and/or intense contrast enhancement pattern on CT scans. Conversely, MRI demonstrated important contrast enhancement in almost all cases. This could be attributed to a higher likelihood of ordering MRI when hypervascularity is suspected on CT.

Regarding the vascularity of ENA-SNT, nearly a quarter of the cases (23.07%) that underwent angiography did not show hypervascularity. Therefore, it is important to emphasize that the absence of hypervascularity should not rule out ENA-SNT. When hypervascularity is present, the external carotid artery (ECA) system is almost always involved, serving as the sole blood supply in 65.38% of cases. There was a contribution from the internal carotid artery (ICA) in 7.69% of cases, and in 3.84%, the ICA was the sole supplier. In JNA, the ECA was reported as the sole blood supply to tumors in 64% of cases. There was a contribution from the ICA in 36% of cases, but it was the sole vascular supply in only 0.48% [65]. 

Histological analysis can confirm the diagnosis of angiofibroma. The angiofibroma tissue consists of a dense fibrous stroma with spindled or stellate cells among a varying amount of collagen fibers and a proliferating vascular component, which presents blood vessels of different sizes. These characteristics are common to both JNA and ENA, often making them indistinguishable histologically. However, Dogan et al. [66] reported that ENAs of the nasal cavity may have some peculiarities, such as an interstitial stromal predominance with fewer vascular elements on histopathologic examination, such as that of long-standing JNA [66–68]. Regarding immunohistochemistry, CD34 was the most frequently found marker in our sample, which is useful to confirm the presence of a vascular tumor. CD34 positivity is not exclusive to angiofibromas and expands the range of differential diagnoses [2, 69]. However, Beham et al. [70] demonstrated that CD34 expression in JNA had an exclusive staining of endothelial cells, while stromal fibroblasts and smooth muscle cells were not reactive. In general, while immunohistochemistry may not be essential for the diagnosis of angiofibroma due to the great variability in marker expressions among tumors [69, 71], it is a valuable tool for differential diagnosis and in distinguishing angiofibromas from other vascular lesions, thus complementing histological examination.

### Differential diagnosis

Diagnosing angiofibromas that originate outside the nasopharynx can be challenging. Because they are extremely rare and have clinical features different from those of JNA, clinical suspicion is low. In many of the reported cases, the diagnosis of ENA-SNT was unexpected, revealed by anatomopathologic examination of surgical specimens. The differential diagnosis includes hemangiomas, hemangiopericytomas, and other vascular malformations [69]. 

### Treatment

Based on the compiled results, complete surgical resection of the tumor can be considered the treatment of choice, demonstrating favorable outcomes, minimal complications, and low recurrence rates. With the popularization of nasal endoscopy, less invasive techniques have become available since 1990, as shown in Fig. 4a. The choice of surgical technique should be tailored to each case, considering both the morbidity and resectability of the tumor. Surgeons should be aware that although ENA-SNTs are less vascular than JNAs, 15.62% of cases showed significant intraoperative or postoperative bleeding. Embolization may be beneficial in the surgical treatment of JNA [72], but its role in ENA-SNT remains unclear due to the lower vascularity of these tumors and the positive surgical outcomes without prior embolization. However, this decision should be individualized, and embolization remains an option for cases where high vascularity is suspected. Although radiotherapy is a treatment option for selected cases of JNA [73], it has rarely been used in the treatment of ENA-SNT and should not be the first choice if the tumor is resectable.

### Recurrence

The recurrence rate was 100% for radiotherapy as first-line treatment and 5.26% for surgery, with a mean time to recurrence of 3.75 months. The recurrence rate for JNA varies in the literature but is often close to 25% [74], with recurrences occurring more frequently within the first 2 years [75, 76]. The lower recurrence rate observed in ENA-SNT can be attributed to factors that facilitate complete tumor resection, such as its less vascular behavior, reduced invasion of adjacent structures, and a more favorable location in less complex anatomical regions.

It is worth noting that the short follow-up period in most of the reports included in this review limits the assessment of long-term recurrence.

### Comparison with previous studies

In the largest review of ENAs published to date, Windfuhr & Vent [2] compiled the characteristics of 174 cases, including tumors across multiple head and neck sites. Compared to ENAs of other sites, our results showed that ENA-SNT has a more rapid progression of symptoms (8.81 vs. 13.1 months), a higher recurrence rate (5.26% vs. 2.3%), a higher risk of biopsy bleeding (44.45% vs. 25%), and a higher frequency of nasal obstruction and epistaxis, similar to that observed in JNA.

### Limitations

This study has limitations inherent in a systematic review of an extremely rare disease, such as small sample size and data collected only from case reports and case series, thus limiting the overall level of evidence. Furthermore, the heterogeneity observed across the reports introduces biases that are intrinsic to this study design.

## Conclusions

The findings of this review confirm ENA-SNT as a clinical entity distinct from JNA. Symptoms develop more rapidly, but the tumor is less aggressive, less vascularized, and has a better prognosis. ENA-SNT can occur across all age groups and in female individuals. Surgical resection is the treatment of choice, with low recurrence rates. Although rare, otolaryngologists should consider ENA-SNT in the differential diagnosis of vascular or indeterminate sinonasal tumors.
